# Supplementary material for: Chained Risk Assessment for Life-Long Disease Burden of Early Exposures–Demonstration of Concept Using Prenatal Maternal Smoking
Source: Int J Environ Res Public Health. 2020 Feb 25;17(5):1472. doi: 10.3390/ijerph17051472 (PMC7084403; doi:10.3390/ijerph17051472)
Supplement: Supplementary file 1 [file ijerph-17-01472-s001.pdf]

Chained risk assessment for life-long disease burden of early exposures – Demonstration of concept using prenatal maternal smoking

SUPPLEMENT

Isabell K. Rumrich [1,2*]; Kirsi Vähäkangas [3]; Matti Viluksela [1,3,4]; Otto Hänninen [2]

[1] University of Eastern Finland (UEF), Department of Environmental and Biological Sciences, Kuopio, Finland
[2] Finnish Institute for Health and Welfare (THL), Department of Public Health Solutions, Kuopio, Finland
[3] University of Eastern Finland (UEF), School of Pharmacy/Toxicology, Kuopio, Finland
[4] Finnish Institute for Health and Welfare (THL), Department of Health Security, Kuopio, Finland

* corresponding author: Email: isabell.rumrich@thl.fi; National Institute for Health and Welfare (THL), Department of Public Health Solutions, P.O. Box 95, 70701 Kuopio, Finland; telephone: +358 29 524 7030

Literature search

Literature review Maternal smoking

Database: PubMed
Date: 24 October 2018
Query: (Smoking[mh] OR tobacco smoke pollution[mh]) AND ((maternal exposure[mh] OR "maternal-fetal exchange"[mh] OR maternal behavior[mh] OR pregnancy[mh] OR "prenatal exposure delayed effects"[mh] OR pregnancy outcome[mh] OR fetal development[mh]) OR ((infant[mh] OR "infant, newborn"[mh] OR mothers[mh] OR female[mh] OR "newborn"[mh]) AND (pregnan* OR in utero OR antepartum OR prenat* OR pre-natal OR peripartum OR peri-natal OR perinat* OR intrapartum OR postnat* OR postpartum OR fetal OR fetus OR foetus OR neonat*)))
Filter: Article Type Meta-Analysis, Review

Literature review preterm birth & Literature review low birth weight

Database: PubMed
Date: 3 October 2018
Query: ((birth) AND (weight OR preterm OR premature)) AND risk) AND meta-analysis

Literature review childhood overweight and obesity

Database: PubMed
Date: 30 November 2018
Query: : ((((child[Title/Abstract] OR children[Title/Abstract] OR childhood[Title/Abstract] OR adolescen*[Title/Abstract] OR teen*[Title/Abstract])) AND (weight[Title/Abstract] OR overweight[Title/Abstract] OR obes*[Title/Abstract] OR BMI[Title/Abstract] OR fat[Title/Abstract] OR (body composition)[Title/Abstract])) AND (risk[Title/Abstract] OR association[Title/Abstract] OR effect[Title/Abstract])) AND meta-analysis[Title/Abstract]

References from the literature review included in BoD estimation

Bhutta AT, Cleves MA, Casey PH, Cradock MM, Anand KJ. Cognitive and behavioral outcomes of school-aged children who were born preterm: a meta-analysis. JAMA. 2002 Aug 14;288(6):728-37.

Burke H, Leonardi-Bee J, Hashim A, Pine-Abata H, Chen Y, Cook DG, Britton JR, McKeever TM. Prenatal and passive smoke exposure and incidence of asthma and wheeze: systematic review and meta-analysis. Pediatrics. 2012 Apr;129(4):735-44. doi: 10.1542/peds.2011-2196. Epub 2012 Mar 19. Review. PubMed PMID: 22430451.

Chu A, Heck JE, Ribeiro KB, Brennan P, Boffetta P, Buffler P, Hung RJ. Wilms' tumour: a systematic review of risk factors and meta-analysis. Paediatr Perinat Epidemiol. 2010 Sep;24(5):449-69.

Cook MB, Akre O, Forman D, Madigan MP, Richiardi L, McGlynn KA. A systematic review and meta-analysis of perinatal variables in relation to the risk of testicular cancer–experiences of the son. Int J Epidemiol. 2010;39:1605–18.

Cortese S, Moreira-Maia CR, St Flur D, Morcillo-Penalver C, Rohde LA, Faraone SV. Association between ADHD and obesity: a systematic review and meta-analysis. Am J Psychiatry. 2016; 173: 34-43.

den Dekker HT, Sonnenschein-van der Voort AMM, de Jongste JC, Anessi-Maesano I, Arshad SH, et al.. Early growth characteristics and the risk of reduced lung function and asthma: A meta-analysis of 25,000 children. J Allergy Clin Immunol. 2016 Apr;137(4):1026-1035.

Gardener H, Spiegelman D, Buka SL. Perinatal and neonatal risk factors for autism: a comprehensive meta-analysis. Pediatrics. 2011 Aug;128(2):344-55.

Hackshaw A, Rodeck C, Boniface S. Maternal smoking in pregnancy and birth defects: a systematic review based on 173 687 malformed cases and 11.7 million controls. Hum Reprod Update. 2011 Sep-Oct;17(5):589-604. doi: 10.1093/humupd/dmr022. Epub 2011 Jul 11. Review. PubMed PMID: 21747128; PubMed Central PMCID: PMC3156888.

Harder T, Rodekamp E, Schellong K, Dudenhausen JW, Plagemann A. Birth weight and subsequent risk of type 2 diabetes: a meta-analysis. Am J Epidemiol. 2007;165:849–57.

Hidayat K, Du X, Shi B-M. Body fatness at a young age and risks of eight types of cancer: systematic review and meta-analysis of observational studies. Obesity Reviews. 2018; 19: 1385-1394.

Huang QT, Gao YF, Zhong M, Yu YH. Preterm Birth and Subsequent Risk of Acute Childhood Leukemia: a Meta-Analysis of Observational Studies. Cell Physiol Biochem. 2016;39(3):1229-38.

Huang J, Zhu T, Qu Y, Mu D. Prenatal, Perinatal and Neonatal Risk Factors for Intellectual Disability: A Systemic Review and Meta-Analysis. PLoS One. 2016 Apr 25;11(4):e0153655.

Lee LJ, Lupo PJ. Maternal smoking during pregnancy and the risk of congenital heart defects in offspring: a systematic review and metaanalysis. Pediatr Cardiol. 2013 Feb;34(2):398-407. doi: 10.1007/s00246-012-0470-x. Epub 2012 Aug 12. Review. PubMed PMID: 22886364.

Li S, Xi B. Preterm birth is associated with risk of essential hypertension in later life. Int J Cardiol. 2014 Mar 15;172(2):e361-3.

Li S, Zhang M, Tian H, Liu Z, Yin X, Xi B. Preterm birth and risk of type 1 and type 2 diabetes: systematic review and meta-analysis. Obes Rev. 2014 Oct;15(10):804-11.

Llewellyn A, Simmonds M, Owen SG, Woolacott N. Childhood obesity as a predictor of morbidity in adultood: a systematic review and meta-analysis. Obesity Reviews. 2016; 17: 56-67.

Mannan M, Mamun A, Doi S, Clavarino A. Prospective associations between depression and obesity for adolescet males and females - A systematic review and meta-analysis of longitudinal studies. Plos One. 2016; 11(6): e0157240.

Mebrahtu TF, Feltbower RG, Greenwood DC, Parslow RC. Childhood body mass index and wheezing disorders: a systematic review and meta-analysis. Pediatr Allergy Immunol. 2015; 26: 62-72.

Mu M, Ye S, Bai M-J, Liu G-L, Tong Y, Wang S-F, et al.. Birth weight and subsequent risk of asthma: a systematic review and meta-analysis. Hear Lung Circ. 2014;23:511–9.

Nicoletti D, Appel LD, Siedersberger Neto P, Guimarães GW, Zhang L. Maternal smoking during pregnancy and birth defects in children: a systematic review with meta-analysis. Cad Saude Publica. 2014 Dec;30(12):2491-529. doi: 10.1590/0102-311X00115813. Review. English, Portuguese. PubMed PMID: 26247979.

Orgel E, Genkinger JM, Aggarwal D, Sung L, Nieder M, Ladas E. Association of body mass index and survival in pediatric leukemia: a meta-analysis. Am J Clin Nutr. 2016; 103: 808-817.

Rayfield S and Plugge E. 2017. Systematic review and meta-analyses of the association between maternal smoking in pregnancy and childhood overweight and obesity. J Epidemiol Community Health, 71: 162-173.

Rosenboom T, de Rooij S Painter R, 2006. The Dutch famine and its long-term consequences for adult health. Early Human Development 82:485-491.

Rumrich IK, Viluksela M, Vähäkangas K, Gissler M, Surcel HM, Hänninen O. Maternal Smoking and the Risk of Cancer in Early Life - A Meta-Analysis. PLoS One. 2016 Nov 8;11(11):e0165040. doi: 10.1371/journal.pone.0165040. eCollection 2016. PubMed PMID: 27824869; PubMed Central PMCID: PMC5100920.

Wang S-F, Shu L, Sheng J, Mu M, Wang S, Tao X-Y, et al.. Birth weight and risk of coronary heart disease in adults: a meta-analysis of prospective cohort studies. J Dev Orig Health Dis. 2014;5:408–19.

Wang C, Geng H, Liu W, Zhang G. Prenatal, perinatal, and postnatal factors associated with autism: A meta-analysis. Medicine (Baltimore). 2017 May;96(18):e6696.

White SL, Perkovic V, Cass A, Chang CL, Poulter NR, Spector T, et al.. Is low birth weight an antecedent of CKD in later life? A systematic review of observational studies. Am J Kidney Dis. 2009;54:248–61.

Wojcik W, Lee W, Colman I, Hardy R, Hotopf M. Foetal origins of depression? A systematic review and meta-analysis of low birth weight and later depression. Psychol Med. 2013;43:1–12.

Yan K, Xu X, Liu X, Wang X, Hua S, Wang C, Liu X. The associations between maternal factors during pregnancy and the risk of childhood acute lymphoblastic leukemia: A meta-analysis. Pediatr Blood Cancer. 2015 Jul;62(7):1162-70. doi: 10.1002/pbc.25443. Epub 2015 Mar 1. Erratum in: Pediatr Blood Cancer. 2016 May;63(5):953-4. PubMed PMID: 25728190.

Table S1.Characteristics of meta-analyses included in this work

	Endpoint	Exposure	Pooled risk estimate	95% CI	# studies	Follow up time	Reference
	Congenital anomalies						
1	Heart	MS	1.11	1.02-1.21	19	na	Lee et al. 2013
2	Oral clefts	MS	1.28	1.2-1.36	38	na	Hackshaw et al. 2011
3	Digestive system	MS	1.18	1.07-1.3	22	na	Nicoletti et al. 2014
4	Musculoskeletal system	MS	1.27	1.16-1.39	48	na	Nicoletti et al. 2014
	Cancer			-			
5	Acute leukemia	PTB	1.09	1.02-1.17	12	<15	Huang et al. 2016a
6	Acute myeloid leukemia	PTB	1.42	1.21-1.67	7	<15	Huang et al. 2016a
7	Acute lymphoblastic leukemia	MS	1.1	1.02-1.19	21	<15	Yan et al. 2015
		OW<br>OB	1.35	1.2-1.51	6	<22 years	Orgel et al. 2016
8	Lymphoma	MS	1.21	1.05-1.36	6	<20	Rumrich et al. 2016
9	Non Hodgkin lymphoma	MS	1.27	1.07-1.48	5	<20	Rumrich et al. 2016
10	Nervous system cancer	MS	1.09	1.02-1.17	22	<20	Rumrich et al. 2016
11	Testicular cancer	LBW	1.34	1.08-1.67	17	na	Cook et al. 2010
12	Wilms' tumor	PTB	1.44	1.14-1.81	6	<15	Chu et al. 2010
13	Oesophageal adenocarcinoma	OW	1.93	1.39-2.68	7	>30	Hidayat et al. 2018
		OB	3.63	1.9-6.89	7	>30	
14	Hepatocellular carcinoma	OW	1.33	1.14-1.54	4	>30	Hidayat et al. 2018
		OB	1.74	1.28-2.32	4	>30	
15	Multiple myeloma	OW	1.24	1.16-1.32	7	>30	Hidayat et al. 2018
		OB	1.53	1.33-1.71	7	>30	
16	Pancreas cancer	OW	1.18	1.12-1.22	10	>30	Hidayat et al. 2018
		OB	1.38	1.24-1.48	10	>30	
17	Renal cell cancer	OW	1.23	1.17-1.29	12	>30	Hidayat et al. 2018
		OB	1.50	1.35-1.66	12	>30	
18	Thyroid cancer	OW	1.13	1.07-1.18	7	>30	Hidayat et al. 2018
		OB	1.26	1.15-1.38	7	>30	
	Cardiovascular						
19	Coronary heart disease	LBW	1.22	1.13-1.31	16	adult	Wang et al. 2014
		OW	1.10	1.07-1.15	4	adult	Llewellyn et al. 2016
		OB	1.21	1.14-1.32	4	adult	
20	Essential hypertension	PTB	1.31	1.2-1.43	8	na	Li et al. 2014a
		OW	1.53	1.33-1.75	2	adult	Llewellyn et al. 2016
		OB	2.28	1.76-2.98	2	adult	
21	Stroke	OW	1.35	1.17-1.56	1	adult	Llewellyn et al. 2016
		OB	1.81	1.36-2.4	1	adult	
	Cognitive						
22	Intellectual disability	PTB	2.03	1.79-2.31	7	na	Huang et al. 2016b
	Mental						
23	Depression in adulthood	LBW	1.15	1-1.32	18	adult	Wojcik et al. 2013
24	Depression	OB	1.4	1.16-1.7	7	11-15 (mean)	Mannan et al. 2016
25	Autism	LBW	1.63	1.19-2.33	15	na	Gardener et al. 2011
		PTB	1.31	1.16-1.48	10	child	Wang et al. 2017
26	Attention Deficit/Hyperactivity Disorder	PTB	2.64	1.85-3.78	7	<15	Bhutta et al. 2002
		OB	1.2	1.05-1.37	30	na	Cortese et al. 2016
	Respiratory						
27	Asthma infant	MS	1.85	1.35-2.53	5	<3 years	Burke et al. 2012
28	Asthma childhood + adolescent	MS	1.23	1.12-1.36	8	5--18	Burke et al. 2012
29	Asthma in childhood	LBW	1.32	1.07-1.62	24	<10	den Dekker et al. 2016
		PTB	1.34	1.15-1.57	24	<10	den Dekker et al. 2016
		OW	1.23	1.17-1.29	28	<10	Mebrahtu et al. 2015
		OB	1.46	1.36-1.57	21	<10	
30	Asthma in adulthood	LBW	1.25	1.21-1.4	4	adult	Mu et al. 2014
	Metabolic						
31	Diabetes	OW	2.41	1.54-3.75	6	adult	Llewellyn et al. 2016
		OB	5.60	2.34-13.3	6	adult	
32	Type 1 diabetes mellitus	PTB	1.18	1.11-1.25	18	all	Li et al. 2014b
33	Type 2 diabetes mellitus	LBW	1.32	1.06-1.64	10	all	Harder et al. 2007
		PTB	1.51	1.32-1.72	5	all	Li et al. 2014b
	Other						
34	Chronic kidney disease	LBW	1.73	1.44-2.08	21	na	White et al. 2009

OR: Odds ratio; MS Maternal smoking; PTB Preterm birth; LBW Low birth weight; OW childhood overweight; OB childhood obesity

Table S2. Endpoints and associated population attributable fractions included in this work

		Background BoD		Maternal smoking			Preterm birth (quit)			Low birth weight (cont)			Low birth weight (quit)			Childhood overweight			Childhood obesity		
**Literature endpoint**	**GBD endpoint**	**DALY**	**95%CI**	**PAF**	**95%CI**	**Reference**	**PAF**	**95%CI**	**Reference**	**PAF**	**95%CI**	**Reference**	**PAF**	**95%CI**	**Reference**	**PAF**	**95%CI**	**Reference**	**PAF**	**95%CI**	**Reference**
**Congenital anomalies**																					
Heart	Congenital heart anomalies	2,653	(2086-3375)	0.01	(0.001-0.01)	Lee et al. 2013															
Oral clefts	Orofacial clefts	240	(155-355)	0.02	(0.014-0.02)	Hackshaw et al. 2011															
Digestive system	Digestive congenital anomalies	2,577	(1777-3555)	0.01	(0.005-0.02)	Nicoletti et al. 2014															
Musculoskeletal system	Congenital musculoskeletal and limb	3,123	(2150-4271)	0.02	(0.011-0.03)	Nicoletti et al. 2014															
**Cancer**																					
Acute leukemia	Acute myeloid leukemia + Acute lymphoid leukemia	806	(664-977)				0.00	(0.001-0.01)	Huang et al. 2016a												
Acute myeloid leukemia	Acute myeloid leukemia	238	(189-294)				0.02	(0.008-0.03)	Huang et al. 2016a												
Acute lymphoblastic leukemia	Acute lymphoid leukemia	568	(475-683)	0.01	(0.001-0.01)	Yan et al. 2015										0.05	(0.03-0.07)	Orgel et al. 2016	0.01	(0.006-0.02)	Orgel et al. 2016
Lymphoma	Hodgkin lymphoma + Non-Hodgkin lymphoma	238	(190-293)	0.01	(0.003-0.02)	Rumrich et al. 2016															
Non Hodgkin lymphoma	Non-Hodgkin lymphoma	191	(158-230)	0.02	(0.005-0.03)	Rumrich et al. 2016															
Nervous system cancer	Brain and nervous system cancer	832	(678-1108)	0.01	(0.001-0.01)	Rumrich et al. 2016															
Testicular cancer	Testicular cancer	11	(7-16)							0.01	(0.002-0.02)	Cook et al. 2010	0.01	(0.002-0.02)	Cook et al. 2010						
Wilms' tumor	Kidney cancer	95	(77-119)				0.02	(0.006-0.03)	Chu et al. 2010							0.03	(0.02-0.04)	Hidayat et al. 2018	0.01	(0.01-0.02)	Hidayat et al. 2018
Oesophageal adenocarcinoma	Esophageal cancer	5,027	(4615-5478)													0.12	(0.05-0.19)	Hidayat et al. 2018	0.07	(0.03-0.15)	Hidayat et al. 2018
Hepatocellular carcinoma	Liver cancer	8,725	(7978-9552)													0.04	(0.02-0.07)	Hidayat et al. 2018	0.02	(0.008-0.04)	Hidayat et al. 2018
Multiple myeloma	Multiple myeloma	5,352	(4661-6495)													0.03	(0.02-0.04)	Hidayat et al. 2018	0.02	(0.01-0.02)	Hidayat et al. 2018
Pancreas cancer	Pancreatic cancer	21,360	(19765-23025)													0.02	(0.02-0.03)	Hidayat et al. 2018	0.01	(0.007-0.01)	Hidayat et al. 2018
Thyroid cancer	Thyroid cancer	1,182	(1053-1324)													0.02	(0.01-0.02)	Hidayat et al. 2018	0.01	(0.004-0.01)	Hidayat et al. 2018
**Cardiovascular**																					
Coronary heart disease	Ischemic heart disease	157,588	147569-172367)							0.01	(0.004-0.01)	Wang et al. 2014	0.01	(0.004-0.01)	Wang et al. 2014	0.01	(0.009-0.02)	Llewellyn et al. 2016	0.01	(0.004-0.01)	Llewellyn et al. 2016
Essential hypertension	Hypertensive heart disease	12,532	(6245-14421)				0.01	(0.008-0.02)	Li et al. 2014							0.07	(0.04-0.09)	Llewellyn et al. 2016	0.04	(0.02-0.06)	Llewellyn et al. 2016
Stroke	Stroke	71,952	(65505-78747)													0.05	(0.02-0.07)	Llewellyn et al. 2016	0.02	(0.01-0.04)	Llewellyn et al. 2016
**Cognitive**																					
Intellectual disability	Idiopathic developmental intellectual disability	1,059	(317-1947)				0.04	(0.03-0.05)	Huang et al. 2016b												
**Mental**																					
Depression in adulthood	Major depressive disorder	292	(242-355)							0.00	(0-0.01)	Wojcik et al. 2013	0.00	(0-0.01)	Wojcik et al. 2013				0.01	(0.005-0.02)	Mannan et al. 2016
Autism	Autism spectrum disorders	4,528	(3095-6269)				0.01	(0.01-0.02)	Wang et al. 2017	0.02	(0.006-0.04)	Gardener et al. 2011	0.02	(0.006-0.04)	Gardener et al. 2011						
Attention Deficit/Hyperactivity Disorder	Attention-deficit/hyperactivity disorder	703	(420-1117)				0.06	(0.03-0.1)	Bhutta et al. 2002										0.01	(0.001-0.01)	Cortese et al. 2016
**Respiratory**																					
Asthma infant	Asthma	0	(0-0)	0.06	(0.02-0.1)	Burke et al. 2012															
Asthma childhood + adolescent	Asthma	2,483	(1522-3695)	0.02	(0.008-0.02)	Burke et al. 2012															
Asthma in adulthood	Asthma	11,326	(8193-15233)							0.01	(0.006-0.01)	Mu et al. 2014	0.01	(0.006-0.01)	Mu et al. 2014						
Asthma in childhood	Asthma	1,065	(604-1698)				0.01	(0.006-0.02)	den Dekker et al. 2016	0.01	(0.002-0.02)	den Dekker et al. 2016	0.01	(0.002-0.02)	den Dekker et al. 2016	0.03	(0.02-0.04)	Mebrahtu et al. 2015	0.01	(0.01-0.02)	Mebrahtu et al. 2015
**Other**																					
Chronic kidney disease	Chronic kidney disease	11,807	(10306-13608)							0.02	(0.01-0.03)	White et al. 2009	0.02	(0.01-0.03)	White et al. 2009						
